# Analgesic Efficacy of Oxycodone in Postoperative Dressings after Surgical Treatment of Burn Wounds: A Randomised Controlled Trial

**DOI:** 10.3390/jcm13030784

**Published:** 2024-01-29

**Authors:** Grzegorz Kowalski, Wojciech Leppert, Małgorzata Domagalska, Monika Grochowicka, Artur Teżyk, Krzysztof Słowiński, Agnieszka Bienert, Danuta Szkutnik-Fiedler, Katarzyna Wieczorowska-Tobis

**Affiliations:** 1 Department of Palliative Medicine, Poznan University of Medical Sciences, 61-701 Pozan, Poland; gkowalski@ump.edu.pl (G.K.); monikagrochowicka@gmail.com (M.G.); kwt@tobis.pl (K.W.-T.); 2 Department of Anesthesiology, Józef Struś Multiprofile Municipal Hospital, 61-701 Poznań, Poland; 3 Department of Palliative Medicine, Institute of Medical Sciences Collegium Medicum, University of Zielona Góra, 65-046 Zielona Góra, Poland; wojciechleppert@wp.pl; 4 University Clinical Hospital in Poznań, Poznan University of Medical Sciences, 61-701 Poznań, Poland; 5 Department of Forensic Medicine, Poznan University of Medical Sciences, 61-701 Poznań, Poland; atezyk@ump.edu.pl; 6 Department of Trauma, Burns and Plastic Surgery, Poznan University of Medical Sciences, 61-701 Poznan, Poland; slowik@man.poznan.pl; 7 Chair and Department of Clinical Pharmacy and Biopharmacy, Poznan University of Medical Sciences, 61-701 Poznań, Poland; agbienert@ump.edu.pl (A.B.); dszkutnik@ump.edu.pl (D.S.-F.)

**Keywords:** burns, opioids, morphine, oxycodone, fentanyl, wounds, pain

## Abstract

Introduction: This study aimed to assess the analgesic efficacy of oxycodone at doses of 10 mg and 20 mg in dressings after surgery of burn wounds. Material and Methods: Twenty adult patients who underwent surgical treatment of third-degree burn wounds under general anaesthesia were included. Burn wounds were treated with dressings, to which oxycodone was added at 20 mg in Group 1 and 10 mg in Group 2. After the surgery, plasma oxycodone and noroxycodone concentrations were assayed, and pain intensity was assessed with Numerical Rating Scale (NRS). Results: In Group 1, no patient reported pain; in Group 2, four patients reported pain. The pain intensity, according to NRS, was 1–8. Plasma concentration of oxycodone in the blood serum was in the range of 1.24–3.15 ng/mL and 1.09–1.28 ng/mL in Group 1 and Group 2, respectively. Noroxycodone was not detected in the plasma. Adverse effects were not observed in any of the treated patients. Conclusions: Oxycodone in dressings provides patients with adequate and safe analgesia.

## 1. Introduction

Annually, approximately 1% of the adult and child population experience burns. Depending on the degree and extent of burns, patients are treated by family doctors in outpatient clinics, stationary surgical departments, and specialist burn treatment units. Burns are the fourth most common type of injury worldwide, after motor vehicle accidents, falls, and physical abuse [1]. Until the first half of the 20th century, treatment for burn patients was limited, and most patients died from shock within the first days after injury. In the second half of the 20th century, regenerative medicine, burn treatment, and pain medicine made significant progress. However, treating burns remains a challenge. Many factors need to be considered when admitting a patient to the ward, such as injury, duration of illness, and tissue involved. Initial assessment of burn sites is often complex and may be associated with poor classification, especially for burns. With deep disease, patients often need surgery to remove irreplaceable tissue. In deep and large-area burns, treatment alone is often insufficient due to the poor condition of the patient [2].

Burns are traumatic injuries that often require a long and challenging recovery. Burns are a part of intensive care and rehabilitation and are often not managed according to patient recommendations. Pain is considered the fifth primary symptom (American Pain Society, 1996) and can result from any injury, including burns. It can be acute or chronic, nociceptive, neurological or psychogenic. Nociceptive pain results from tissue damage, neuropathic pain from nerve damage, and psychogenic pain from psychological factors [3]. Burns are unique due to their painful, neuropathic, inflammatory, and nociceptive properties [4]. In addition to causing death, burn injuries can cause disfigurement, prolonged hospital stay, physical and mental stress, and suicide compared to other types of injuries. Inadequate pain management that does not meet the needs of burn patients often leads to opioid overdose and opioid dependence. The current opioid abuse crisis in the United States has caused physicians to re-examine expectations and strategies [5].

In Poland, in five specialist units, several thousand patients are treated annually. However, there is a lack of detailed statistical data. At the Department of Trauma, Burns, and Plastic Surgery in Poznań, approximately 500–800 surgical procedures are conducted annually in patients who experienced burn trauma. The treatment is long-term and often requires several surgical interventions. Wound care is excruciating for patients and involves the introduction of adequate and effective analgesia. Despite the use of available analgesics, according to WHO, including opioids and adjuvants, their efficacy is still too low. Patients often experience concerns and anxiety when entering an operation theatre again.

The study assessed the analgesic effectiveness of 20 mg and 10 mg of oxycodone in dressings used after surgery on burn wounds. The null hypothesis was that the severity of pain would not differ among the patients’ groups.

## 2. Materials and Methods

**Figure 1:**
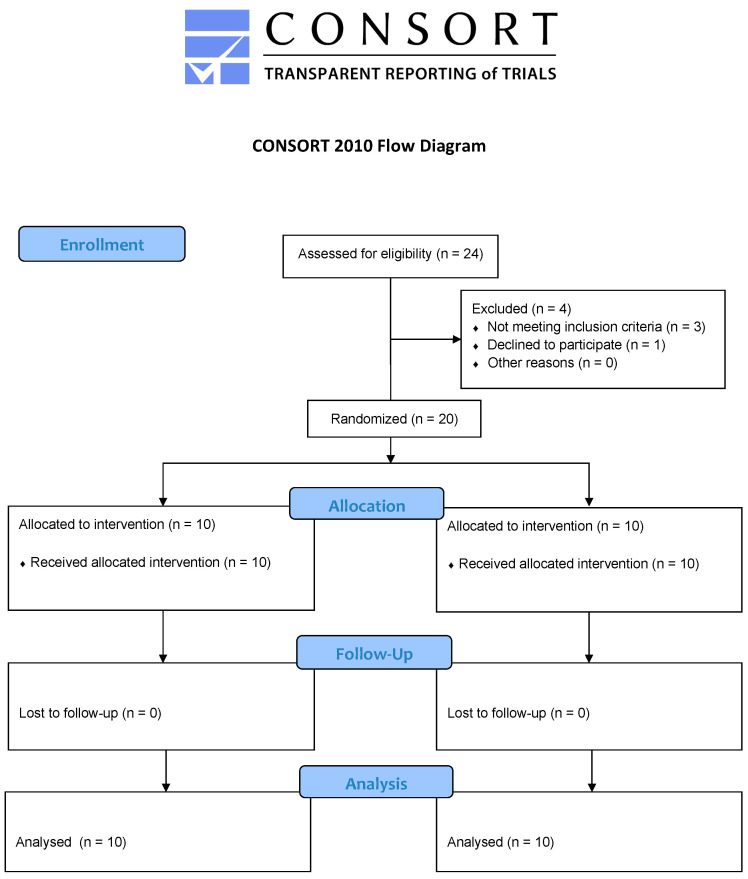
Flowchart of the study.

According to the Good Clinical Practice Guidelines (GCP) and the Declaration of Helsinki, this was a prospective, double-blinded, randomised controlled trial in two parallel groups. Our Institutional Reviewer Board reviewed and approved the trial (protocol number 887/15) and registered it with clinicaltrails.gov (NCT06142591). We followed the Consolidated Reporting Trials Standards (CONSORT), as shown in Figure 1.

Enrollment occurred from the 1st of January 2016 to the 14th of June 2018. Twenty adult patients with fire third-degree burns on the chest and the abdomen, treated at the Surgery and Rehabilitation Clinic in Poznań, Poland, were included in this study. Enrolment was offered before surgery to adults anticipated for debridement of burn wounds without skin graft amputation. Patients who refused to take part in the study, mechanically ventilated patients, or patients with poor mental performance were restricted from the survey. Written informed consent was obtained from all patients for this research program. Using computer software nQuery Advisor (version 7.0, Statistical Solutions, Boston, MA, USA), patients were randomly assigned to receive 10 or 20 mg of oxycodone wound dressing. Research participants were conscious, fully communicated, and familiar with clinical conditions and procedures; all agreed to participate in this study. This study covers burn wounds up to 24 h after surgery.

The double-blindness of the study was conducted by creating a task for researchers who did not know all the final scores. The first researcher, uninvolved in the study, prepared random names and kept the study groups in opaque, sealed envelopes. Another anesthesiologist followed the lead, quickly opening the envelope before dressing to inform the surgical team and performing the procedure as directed. Therefore, the research group was unaware of the patient, surgeon, operating room personnel, and anaesthesia team. After the data analysis was completed, group unblinding was performed. 

During the surgery, patients underwent general anaesthesia. For the induction of anaesthesia, the following drugs were used iv: propofol (2 mg/kg) and fentanyl (100–200 μg). Patients were put on a laryngeal mask, and anaesthesia was continued using the following gas mixture: anaesthetics—sevoflurane (MAC 0.5–1.0), and oxygen and air (50/50) in the assisted mode—by volume/pressure variable or spontaneous. During anaesthesia, fentanyl (50–100 μg iv) was added every 20–30 min. At the end of the surgery, patients received ketoprofen 100 mg iv. After surgery, patients received pain treatment: ketoprofen 100 mg iv twice daily (at 8 pm and 8 am). If the Pain Rating Scale (NRS) number was more significant than 4, 5 mg of morphine sulfate was administered. 

After the debridement of the third-degree burns, the wound was closed with three layers of sterile dressing. The first dressing layer was wetted with octenidine and phenoxyethanol with oxycodone (Oxynorm, Mundipharma, Tokyo, Japan), the second dressing layer was moistened with paraffin, and the third layer was dried. Group 1 received 20 mg of oxycodone plus octenidine and phenoxyethanol in the dressings. Group 2 received 10 mg of oxycodone plus octenidine and phenoxyethanol in the wound dressing.

During the surgery, demographic data, age, gender, weight, height, American Society of Anesthesiologists (ASA) physical status classification system, duration of surgery, fentanyl consumption, degree of burn, per cent of burn surface with wound surface after possible skin collection, and volume of octenidine used were assessed and measured. The dressing concentration of oxycodone was measured before the application of wound dressing. After the surgery to conduct laboratory tests to assay the level of oxycodone and noroxycodone in the blood serum in each patient, a sample of 4 mL of peripheral blood was collected five times at the following time points: 1, 2, 3, and 6 h since the completion of surgery and 5th time at the moment of reporting pain. The time was determined, and pain intensity was assessed using the Numerical Rating Scale (NRS) at each blood sample collection. Patients were monitored for adverse effects such as dizziness, nausea, vomiting, drowsiness, shallowing breath, and a reduced respiratory rate.

### Statistics

To estimate the sample size, we analysed our hypothesis that oxycodone dressing upgrades pain management. While there were no analogous clinical studies, we predicted pain severity with an index of 0 and a standard deviation of 1 based on published data on the use of morphine for pain management. We simulated drift using a truncated Gaussian distribution of 0 to 10, SD of 1, and mean of 0 (for the 10 mg oxycodone group). We imitated ten subjects in each group with these hypotheses, and both sides = 5%. With a total sample size of twenty patients, we evaluated a power of 70% to discover the difference in pain among groups of less than 1. For the statistical analysis, we used the GraphPad Prism 8 software program (verison 10.1.1 (270), GraphPad Software Inc., San Diego, CA, United States).

The Kołmogorov–Smirnov normality test was used to estimate the parametric distribution of numerical variables. Differences between groups were evaluated with the t-Student or Mann–Whitney U test. Categorical variables were assessed with the Mann–Whitney U test, and statistical analyses were evaluated with Fisher’s test. *p* value < 0.05 was considered significant.

## 3. Results

Three of the 24 patients were postponed for qualification, three did not fit the inclusion criteria, and one refused to participate in the clinical trial. The remaining 20 participants were randomly assigned to one of the two groups, as shown in Figure 1. There were no clinically relevant differences in the characteristics of the groups, as shown in Table 1.

Therefore, the study outcomes are summarised in Table 2 and Table 3.

In Group 1, three patients were classified as ASA III due to epilepsy and advanced hypertension. One patient from Group 1 was classified ASA II due to alcohol abuse. In Group 2, four patients were classified as ASA III due to advanced hypertension. One was classified as ASA II due to periodical pain in the spine. The rest of the patients in both groups were classified as ASA I. Among all patients, the range of the percentage of skin surface area associated with the burn, including harvested skin grafting, was 13.7 ± 4.0% in group 1 and 12.7 ± 4.5% in group 2, as seen in Table 1. The wound localisation did not differ between groups, with *p* = 0.8425.

In all patients, after applying a wound dressing soaked in a solution of octanidine + phenoxyethanol containing oxycodone, there was no need to administer fentanyl until the end of the operation. Moreover, all patients could breathe the anaesthetic gases mixture (air, oxygen, and sevoflurane) independently.

The mean oxycodone concentrations in the dressing were higher in group 1 (0.0530 ± 0.0095 mg/mL) than in group 2 (0.0290 ± 0.0081 mg/mL; *p* < 0.0001). Also, serum oxycodone concentration levels were not statistically different between the two groups at all time points, as shown in Table 2.

The most crucial is that noroxycodone concentration levels were 0 ± 0 ng/mL at all time points in both groups.

In group 1, the pain score (NRS) was 0 at 1, 2, and 3 h following the operation in every patient. Six hours after surgery—two patients reported pain with NRS scores 1 and 2; the dressing oxycodone concentration levels were 0.05 and 0.04, respectively.

In group 2, one subject stated pain twice, with pain scores (NRS) of 5 and 8, in hours 1 and 3, respectively, after the burn operation; the serum level of oxycodone was 1.18 ng/mL and 1.09 ng/mL, respectively, and the dressing concentration level was 0.025 mg/mL. Two subjects were notified of pain once, with pain scores (NRS) of 5 and 6. In both cases, the serum concentration levels were 0.03 mg/mL. The pain scores expressed in NRS in the remaining subjects were 0 at all time points.

All along the trial period (up to 24 h after surgery), no systemic or local adverse effects—such as breathing difficulties, nausea, vomiting, dizziness or sedation, and itching, tingling, and numbness—were reported in any participants.

## 4. Discussion

Annually, approximately 1% of the population experience burns. Depending on the degree and extent of burns, patients are treated by family doctors in outpatient clinics, stationary surgical departments, or specialist burns treatment units.

Burn trauma and burn wounds are the most challenging and longest-healing traumas [6]. Consequences and the severity of the treatment depend on the degree and extent of burns; they can be from mild, which does not require physicians’ intervention, to very severe, including death in their course [7,8,9]. Patients may experience electrolyte imbalance, metabolic disturbances, lowering protein level, loss of body weight, and infection complications—local concerning burn wounds or (because of immunosuppression induced by the trauma) generalised infections and sepsis with multiorgan disturbances, renal, heart, and respiratory failure—which may lead to death [10]. Due to the suddenness and severity of the trauma course, patients often experience posttraumatic stress accompanied by anxiety or depression.

Systematic opioid analgesics have been considered the standard treatment for burn pain for many years. The overwhelming consensus is that opioid therapy is an essential tool in the treatment of burn wound pain. In a retrospective, multi-institutional study, Sheridan et al. [11] reported that increased opioid use in the early stages of burn treatment was associated with increased severity and rate of medium- and long-term posttraumatic stress disorder. Persistent burning pain causes burn patients to develop tolerance, which may affect the effectiveness of opioids in this population over time. On the other hand, Wibbenmeyer et al. [12] showed that increased opioid use was associated with increased pain and need for opioids, even after controlling for burn severity, preoperative pain scores, and other variabilities. The authors conclude that this has to do with tolerance for opioids. Finally, many authors have described the phenomenon of opioid-induced hyperalgesia in burn patients [10,13,14,15,16]. Interestingly, many doctors use methadone to prevent tolerance and/or hyperalgesia [14,17,18]. However, the use of methadone in burn patients is limited to retrospective studies [19]. The data clearly show that opioid use requires careful assessment of the patient’s tolerance and ongoing titration of these medications, as the reason varies in different patients [8,9,20,21,22]. In burn patients, pharmacogenetic polymorphism has been clearly associated with differences in opioid use [23,24]. Additionally, metabolic and fluid changes associated with severe thermal injury can lead to significant changes in the volume of distribution and pharmacokinetics of opioids and burn injury [23]. Most of the information about the effectiveness and safety of opioids in burn patients comes from studies using opioids to treat surgical pain during dressing. Systematic opioids are standard practice for treating burn injuries and pain in most of the centres in North America and Europe [25,26].

Fentanyl is a safe and effective medication for pain management and burn replacement therapy in adults and children. Intravenous fentanyl infusion is often used for close monitoring due to the risk of conscious sedation [23]. Because the intravenous fentanyl requirements vary from patient to patient and cannot be reliably determined based on patients’ targets, such as burns or age, the dose must be carefully titrated for each patient. To accommodate the rapid onset of pain associated with burn treatment, various opioid analgesics have been used to provide faster induction of anaesthesia [27,28,29]. Safe and effective burn dressing analgesia was achieved in non-intubated patients using propofol and sufentanil in the target infusion system Pole [24,30]. Remifentanyl is safe and effective, both as an adjunct to propofol and as a monotherapy [24,30]. Alfentanil is effective in several studies, whether as a target infusion or by patients [14,31].

Topical opioids are thought to provide local pain relief by binding to nearby peripheral opioid receptors that cause pain [32,33]. Topical opioid use, in general, is an understudied topic, especially in burn wound treatment. However, several trials studied topical opioids in the treatment of malignant fungating wounds [33], where topical opioids, especially oxycodone, were found to relieve pain.

The treatment is complex and multidisciplinary and requires the involvement of medical doctors, behaviourists, psychologists, and physiotherapists [1,2,3,12]. It comprises the period of stabilisation of organism functioning, rehabilitation, and sometimes recurring or chronic symptoms, which persist for many years, such as neuropathic pain including stinging, tingling, and itch, often with anxiety and depression [4,10,13]. During the treatment, necrotic tissues must be removed and supplied with moist dressings with antiseptics as soon as possible. This study used octenidine dressings, which decreased pain intensity and improved the treatment’s efficacy, shortening the treatment time compared to formerly used dressings with vaseline or iodine [5].

There is a continued search for new solutions to achieve better effects, e.g., silver, nitric oxide, or different types of honey [11]. Among many observed local and systemic adverse effects, a significant problem is pain appearing during and after trauma and during the treatment. Pain intensifies negative phenomena in the organism, ischemia, and oedema of tissues damaged during the trauma, potentiates systemic symptoms, and may lead to a patient’s death. Pain appearing after a burn makes it very difficult to obtain relief. It requires using a full spectrum of drugs, according to the WHO analgesic ladder, along with adjuvant analgesics, and often, this does not provide adequate analgesia [10].

The local use of opioids provides many open wounds with unexpectedly sound effects, improving treatment efficacy [13,14,15,16,17]. Despite systemic administration (iv, sc, po) of strong analgesics after surgical treatment of burn wounds, patients restrict mobility in the trauma area because each movement induces and intensifies pain. The lack of movement intensifies the disturbance of local circulation and tissue ischemia. Ischemic and hypoxic tissues display worse healing and constitute a good base for bacteria colonisation and local and secondarily systemic infection development [6,34,35,36]. In this study, patients after wound surgery and the first dressing application with oxycodone (in both groups) until the completion of surgery did not require additional doses of fentanyl and could remain on their breath with an anaesthetic gas mixture used. They quickly woke up without pain and could freely move contracting muscles around tissue trauma without pain. Their movements were only limited by a dressing. Oxycodone concentrations of 0.04–0.06 in the disinfectant liquid induced a painless postoperative course during the first day after the surgery. Both doses resulted in the painless waking up of patients. What is very important is that oxycodone is practically not absorbed into the bloodstream after being applied to a burn wound. Oxycodone blood concentrations are clinically insignificant, and oxycodone metabolite noroxycodone is undetectable in blood.

Even when using systemically high opioid doses, such sound effects were not observed [7,37,38,39]. The oxycodone in plasma serum was only detectable in single samples, and oxycodone concentration did not exceed a few ng/mL. Their amounts could not be accountable for the pain management effects observed [38,40]. Values of oxycodone concentration providing analgesia after IV oxycodone administration are ten times higher [41]. This suggests a receptor mechanism of opioid action with probable peripheral blocking of neurosensitisation of the central nervous system (patients after administration of the first dressing did not require additional fentanyl doses) [42,43].

Local use of opioids renders unexpectedly sound effects regarding analgesia and benefits in terms of accelerating wound healing [14,15,16,17,19,31,44]. Opioid receptors are located in the area of open wounds, injuries in the course of cancer, and ulcerations [29,36,45]. The mechanism of opioid action is blocking receptors located at endings of peripheral sensory nerves, immune cells, and cutaneous cells, blocking receptors present at infected tissues (skin grafts, arthritis, chronic wounds, and burns)—a result of local action and inhibition of delivery of pro-inflammatory neuropeptides and falling inflammation [43,44]. Morphine is the most frequently used opioid in 1% gel [46], less often meperidine (100 mg water solution with xylocaine), methadone (100 mg in 10 g powder), oxycodone (5 mg in 1–2 mL water solution), or medical cannabis. Sound effects were observed after local administration of ibuprofen, diclofenac, ketamine, capsaicin, and lidocaine in dressings [18,19,23,24,30,31,32,33,47,48,49].

Opioids administered topically stimulate angiogenesis through activation of endothelium cells to proliferation and formation of new vessels (revascularisation), which is correlated with improved oxygenation, blood supply, and tissue nourishment, stimulating lymphogenesis and nerve regeneration, which leads to faster wound healing [41,50,51]. After local administration of opioids, no drowsiness, vertigo, or other symptoms associated with systemic administration of opioids have been observed.

Further studies are needed to investigate the physiological and clinical mechanism of opioid tolerance and opioid-induced hyperalgesia in burn patients. A broader definition of the pathophysiology that causes these conditions is needed to improve such studies. In the context of the national opioid epidemic, there is an urgent need to determine the relationship between use for pain and the development of pain, chronic and/or opioid abuse. Strategies should be developed to prevent severe damage to the healing process of burn injuries.

The main limitation of this study was its small sample size. Another limitation was that we did not obtain blood samples and NRS scores after 6 h following surgery. Despite these limitations, this study has several critical original contributions to the literature and significant implications for clinical practice. This is the first application of oxycodone in burn wound dressing. It shows that oxycodone applied in dressing does not achieve clinically significant blood concentration levels. Thus, oxycodone applied in wound dressing may be a safe alternative to systematic opioids, especially in the context of the pandemic postoperative opioid overdose across North America and Europe.

## 5. Conclusions

Oxycodone administered in dressings at a dose of 20 mg (in concentrations 0.04–0.06 ng/mL) prevents pain appearance, and an amount of 10 mg (in concentrations 0.02–0.05 ng/mL) significantly limits pain appearance. Topical administration of oxycodone in single cases is associated with getting the drug into the blood. However, in a clinically insignificant amount, it binds to peripheral opioid receptors and probably blocks central sensitisation in the CNS. Topical administration of oxycodone is an effective and safe method of pain management after surgical treatment of burn wounds and beneficially influences the healing process. The oxycodone in postoperative dressings provides adequate and safe analgesia.

## Figures and Tables

**Table 1:** Baseline characteristics of Group 1 and Group 2. Values are mean (SD) or number.

	Group 1 (20 mg Oxycodone) n = 10	Group 2 (10 mg Oxycodone) n = 10	*p*-Value
ASA	2.1 (SD = 0.7)	2.2 (SD = 0.6)	0.7486
Comorbidities:			
Hypertension	3	5	0.6499
Alcohol abuse	2	3	>0.9999
Age (years)	45.6 (SD = 15.7)	541.2 (SD = 17.1)	0.9556
Sex (F/M)	4/6	3/7	0.9999
Height (cm)	171.8 (SD = 7.5)	173.0 (SD = 9.7)	0.5392
Weight (kg)	89.3 (SD = 8.4)	80.9 (SD = 15.2)	0.2542
Wound surface (%)	13.7 (SD = 4.0)	12.7 (SD = 4.5)	0.5136
Wound localisation:			
Chest	3	4	
Abdomen	3	2	
Chest and abdomen	4	4	0.8425
The volume of octanidol (mL)	346.0 (SD = 79.9)	395.0 (SD = 103.9)	0.1787
FN received during surgery (mcg)	244 (SD = 39.5)	301.0 (SD = 146.5)	0.3606
Surgery duration (min)	97.00 (SD = 38.2)	94.5 (SD = 42.5)	0.9272

ASA—American Society of Anesthesiologists physical status classification system, F—female; M—male; cm—centimeters; kg—kilograms; mL—milliliters; mcg—micrograms; min—minutes.

**Table 2:** Serum oxycodone and noroxycodone concentration levels (ng/mL) and NRS score in both groups.

Patients		Postoperative												Dressing Oxycodone Concentration mg/mL
		1 h			2 h			3 h			6 h			
		SOC	SNC	NRS	SOC	SNC	NRS	SOC	SNC	NRS	SOC	SNC	NRS	After Surgery
**Group 1**	**1**	0	0	0	0	0	0	0	0	0	0	0	0	0.06
	**2**	0	0	0	0	0	0	0	0	0	0	0	0	0.06
	**3**	1.78	0	0	2.15	0	0	1.56	0	0	1.24	0	0	0.06
	**4**	1.72	0	0	3.15	0	0	1.12	0	0	0	0	0	0.04
	**5**	0	0	0	0	0	0	0	0	0	0	0	0	0.06
	**6**	0	0	0	0	0	0	0	0	0	0	0	0	0.06
	**7**	1.82	0	0	2.43	0	0	1.77	0	0	1.64	0	2	0.05
	**8**	0	0	0	0	0	0	0	0	0	0	0	1	0.04
	**9**	0	0	0	0	0	0	0	0	0	0	0	0	0.06
	**10**	0	0	0	0	0	0	0	0	0	0	0	0	0.04
**Group 2**	**1**	0	0	0	0	0	6	0	0	0	0	0	0	0.03
	**2**	0	0	0	0	0	0	0	0	0	0	0	5	0.03
	**3**	0	0	0	0	0	0	0	0	0	0	0	0	0.03
	**4**	0	0	0	0	0	0	0	0	0	0	0	0	0.05
	**5**	0	0	0	1.28	0	0	0	0	0	0	0	0	0.025
	**6**	1.25	0	0	0	0	0	0	0	0	0	0	0	0.02
	**7**	0	0	0	0	0	0	0	0	0	1.16	0	0	0.025
	**8**	0	0	0	0	0	0	0	0	0	0	0	0	0.025
	**9**	0	0	0	0	0	0	0	0	0	0	0	0	0.03
	**10**	1.18	0	5	0	0	0	0	0	8	1.09	0	0	0.025

SOC—serum oxycodone concentration; SNC—serum noroxycodone concentration.

**Table 3:** Study outcomes. Values are mean (SD or minimum and maximum) or numbers.

	Group 1 (20 mg Oxycodone) n = 10	Group 2 (10 mg Oxycodone) n = 10	*p*-Value
**NRS postoperative**			
1 h	0 (0–0)	0 (0–0)	1.0
2 h	0 (0–0)	0.9 (0–6)	0.9999
3 h	0 (0–0)	0.8 (0–8)	0.9999
6 h	0 (0–0)	0.5 (0–5)	0.9999
**Serum oxycodone concentration levels (ng/mL)**			
1 h	0.5320 (SD = 0.8569)	0.2430 (SD = 0.5126)	0.3731
2 h	0.7730 (SD = 1.268)	0.1280 (SD = 0.4048)	0.2105
3 h	0.4450 (SD = 0.7334)	0.0000 (SD = 0.0000)	0.2105
6 h	0.2880 (SD = 0.6144)	0.2250 (SD = 0.4746)	0.7214
**Serum noroxycodone concentration levels (ng/mL)**			
1 h	0 (0–0)	0 (0–0)	1.0
2 h	0 (0–0)	0 (0–0)	1.0
3 h	0 (0–0)	0 (0–0)	1.0
6 h	0 (0–0)	0 (0–0)	1.0
**Dressing oxycodone concentration (mg/mL)**			
After surgery	0.0530 (SD = 0.0095)	0.0290 (SD = 0.0081)	<0.0001

mL—milliliters; mcg—micrograms; ng—nanograms.

## Data Availability

The study datasets are available from the corresponding author at reasonable request.
